# Effects of whole-body vibration training on inflammatory markers and lipid profiles in adults: a systematic review and meta-analysis of randomized controlled trials

**DOI:** 10.3389/fimmu.2025.1705866

**Published:** 2025-11-10

**Authors:** Yan Pan, Ping Luo, Junpeng Pang

**Affiliations:** 1 School of Sports Training, Wuhan Sports University, Wuhan, China; 2 School of Physical Education, Wuhan Sports University, Wuhan, China

**Keywords:** whole-body vibration, vibration training, inflammation, lipid profile, cardiovascular disease

## Abstract

**Objective:**

The aim of this study was to synthesize existing randomized controlled trials (RCTs) to investigate the effects of whole-body vibration (WBV) training on inflammatory markers and lipid profiles in adults.

**Methods:**

In this meta-analysis, we systematically searched four major electronic databases—PubMed, Web of Science, Cochrane, and Embase—from their inception until August 15, 2025, to ensure comprehensive coverage of the relevant literature. The primary outcome indicators analyzed in this study included interleukin-6 (IL-6), interleukin-8 (IL-8), tumor necrosis factor-alpha (TNF-α), high-sensitivity C-reactive protein (hs-CRP), triglycerides (TG), total cholesterol (TC), low-density lipoprotein (LDL), and high-density lipoprotein (HDL). We conducted data analysis using the Stata 17.0 software to perform a statistical analysis of the combined effect sizes for each indicator.

**Results:**

A total of 11 RCTs were included, involving 409 participants. The results showed that WBV training significantly reduced TNF-α [weighted mean difference (WMD) = −0.80 pg/mL, 95% CI: −1.14 to −0.47, p < 0.001], hs-CRP (WMD = −0.68 mg/L, 95% CI: −0.94 to −0.43, p < 0.001), and TG [standardized mean difference (SMD) = −0.67, 95% CI: −1.15 to −0.19, p = 0.006] levels in adults. However, no significant effects were observed for IL-6 (WMD = −0.50 pg/mL, 95% CI: −2.08 to −1.08, p = 0.535), IL-8 (WMD = 0.30 pg/mL, 95% CI: −0.26 to 0.85, p = 0.296), TC (SMD = −0.28, 95% CI: −0.57 to 0.02, p = 0.067), LDL (SMD = −0.47, 95% CI: −0.98 to 0.03, p = 0.068), and HDL (SMD = 0.43, 95% CI: −0.17 to 1.03, p = 0.156).

**Conclusion:**

This study confirmed that WBV training can positively impact inflammatory markers (TNF-α and hs-CRP) and lipid profile (TG) in adults. These findings provide important evidence for health management and intervention strategies for chronic disease prevention in adults, further supporting WBV training as an effective exercise intervention strategy for promoting metabolic health.

**Systematic Review Registration:**

PROSPERO (CRD420251124521) https://www.crd.york.ac.uk/prospero/CRD420251124521.

## 1 Introduction

Cardiovascular disease (CVD) is a leading cause of death worldwide (1, 2). Increasing evidence suggests that chronic inflammation can affect cardiovascular health through various mechanisms, thereby increasing the risk of hypertension, diabetes, atherosclerosis, and other forms of CVD (3, 4). Meanwhile, abnormalities in lipid profiles are also considered important markers for the occurrence of CVD, particularly the elevation of low-density lipoprotein (LDL) and the reduction of high-density lipoprotein (HDL), both of which are closely associated with an increased risk of cardiovascular events (5). Currently, drug treatments are widely used to lower inflammatory responses and improve lipid profiles to reduce the incidence and mortality of CVD (6, 7). Despite the significant effects of pharmacological treatments in reducing CVD risk, they still face challenges such as drug side effects, adherence issues, and difficulties in accommodating individual differences (8). In contrast, lifestyle interventions are considered an effective and sustainable strategy for significantly improving cardiovascular health (9).

Exercise, especially aerobic exercise, has been extensively proven through numerous studies to significantly enhance individual physical health and overall well-being (10–16). However, approximately one-third of adults worldwide do not meet the recommended exercise guidelines (17). Many individuals face various barriers during prolonged aerobic exercise—such as limited time, lack of motivation, and difficulty in engaging those with mobility challenges—which often hinder their continuous participation (18, 19). In contrast, whole-body vibration (WBV) training, as a unique form of exercise, involves placing individuals on a vibration platform in specific positions (such as sitting or standing) to transmit vibrational stimuli throughout the body and induce physiological effects (20, 21). This training method effectively enhances muscle strength (22), improves blood circulation (23), increases balance abilities (24), and also provides an alternative option for those who find it challenging to engage in traditional aerobic exercise, thus helping them overcome exercise barriers and improving overall health and quality of life (25, 26). Although WBV training has been confirmed by numerous meta-analyses to enhance muscle strength, improve gait and balance, and increase quality of life (27–29), there is currently a lack of systematic reviews and quantitative evidence to clearly assess the effects of WBV training on inflammatory markers and lipid profiles. Current research has shown that WBV training can significantly increase the levels of biomarkers such as nitric oxide (NO), adiponectin, and irisin in the body (30–32). These substances play a critical role in regulating metabolism, promoting blood circulation, and reducing inflammatory responses, suggesting that WBV training may positively impact the improvement of inflammatory markers and lipid profiles. Considering that inflammatory markers and lipid profiles are closely related to the development of various CVDs and are regarded as important risk factors, exploring the effects of WBV training in these areas has significant clinical and public health implications (33). This could provide an effective alternative for individuals who find it difficult to engage in traditional exercise, helping them reduce the risk of CVD and improve their quality of life.

Therefore, this study aimed to conduct a systematic review and meta-analysis to integrate data from existing randomized controlled trials (RCTs) and evaluate the effects of WBV training on inflammatory markers and lipid profiles in adults, providing evidence-based support for clinical practice. We hypothesize that WBV training can significantly improve inflammatory biomarkers and lipid profile levels in adults, thereby exerting a positive impact on overall health.

## 2 Methods

### 2.1 Protocol registration

This systematic review and meta-analysis was conducted strictly following the Preferred Reporting Items for Systematic Reviews and Meta-Analyses (PRISMA) to ensure the transparency and credibility of the research (34). Additionally, this study was registered on the PROSPERO official platform, with registration number CRD420251124521.

### 2.2 Literature search strategy

A comprehensive search was conducted across four electronic databases—PubMed, Cochrane Library, Embase, and Web of Science—covering the time frame from the inception of the databases until August 15, 2025. By screening subject terms in PubMed, Cochrane Library, and Embase, relevant free-text terms associated with these subject words were obtained. The specific search terms used were as follows: “whole-body vibration training” OR “whole body vibration training” OR “vibration training” OR “vibration” OR “WBVT” AND “inflammation” OR “inflammatory response” OR “inflammatory markers” OR “interleukins” OR “IL” OR “tumor necrosis factor-alpha” OR “TNF-alpha” OR “TNF-α” OR “C-reactive protein” OR “CRP” OR “high sensitivity C-reactive protein” OR “hs-CRP” OR “lipids” OR “lipid profile” OR “total cholesterol” OR “TC” OR “triglycerides” OR “TG” OR “lipoproteins” OR “low-density lipoprotein” OR “LDL” OR “low-density lipoprotein cholesterol” OR “LDL” OR “LDL-C” OR “high-density lipoprotein” OR “HDL” OR “high-density lipoprotein cholesterol” OR “HDL cholesterol” OR “HDL-C” AND “random” OR “randomized” OR “randomly” OR “randomized” OR “randomized controlled trial” OR “RCT” OR “randomized controlled trials” OR “RCTs”. Two senior researchers (YP and PL) independently conducted the literature search using the same search terms. They also screened relevant review articles to ensure the comprehensiveness of the literature search. The literature search process covered four electronic databases, with detailed information provided in **Supplementary Material 1**.

### 2.3 Literature inclusion and exclusion criteria

#### 2.3.1 Inclusion criteria

Studies were included if they met all of the following criteria: 1) adults of any health status (aged ≥18 years); 2) to eliminate other potential confounding factors, the experimental group received only WBV training (excluding combined interventions involving WBV training with other training and dietary modifications). Meanwhile, the control group did not undergo any training or receive only standard care and light exercise; 3) studies must include common inflammatory markers and lipid profile data collected before and after the intervention; 4) the intervention period must be ≥2 weeks; and 5) studies must be RCTs.

#### 2.3.2 Exclusion criteria

Studies were excluded if they met any of the following criteria: 1) the experimental group received WBV training in combination with any other intervention, 2) animal studies, 3) non-RCTs, 4) acute experiments, or 5) non-English publications.

### 2.4 Risk of bias and quality assessment

The Cochrane Risk of Bias Assessment Tool (RoB 2) was used to evaluate the risk of bias in the included RCTs (35). This tool comprises five main assessment domains, with each domain’s risk of bias classified as “low risk”, “high risk”, or “some concerns”. By analyzing each criterion, an overall conclusion regarding the risk of bias will be drawn, providing significant evidence for the credibility of the research.

Additionally, the Physiotherapy Evidence Database (PEDro) scale was utilized to assess the quality of the included studies (36, 37). The PEDro scale contains 11 items aimed at comprehensively evaluating the design, implementation, and reporting quality of clinical trials. Each item is scored as “1” for meeting the criteria or “0” for not meeting the criteria, determining the risk of bias in the study. Based on the scores, studies are classified as high quality (score ≥ 6), fair quality (score 4 to 5), or low quality (score < 4). Due to the difficulties of implementing blinding for subjects and researchers in exercise-related RCTs, we did not evaluate the blinding items in the PEDro scale.

To ensure the reliability of the risk of bias and quality assessment results in the included studies, the entire evaluation process was conducted independently by two senior researchers (YP and PL). In cases of any discrepancies during the assessment, discussions and consultations with a third researcher (PJP) were conducted to ensure the consistency and accuracy of the evaluation results.

### 2.5 Data extraction

Two experienced researchers (YP and PL) independently extracted literature information using the same Excel spreadsheet. The extracted content included basic information such as author names, publication year, study design, sample size, intervention measures, and primary outcome indicators. During the data extraction process, both researchers strictly adhered to the predefined spreadsheet and standard operating procedures to ensure the standardization and consistency of the data. All extracted data were cross-checked to ensure accuracy and completeness. The outcome indicator data were converted to mean and standard deviation using the methods provided by Cochrane. The formula for converting means is as follows:


$$MD_{diff}=M_{post}-M_{pre}$$

Here, M_post_ and M_pre_ represent the mean values of baseline and post-intervention outcome indicators, respectively. The formula for converting SD is as follows:


$$\sqrt{\left(\text{S}{\text{D}}_{\text{pre}}^{2}+\text{S}{\text{D}}_{\text{post}}^{2}\right)-\left(2\times \text{Corr}\times \text{S}{\text{D}}_{\text{pre}}\times \text{S}{\text{D}}_{\text{post}}\right)}$$

The correlation coefficient (Corr) was set to 0.5 according to the Cochrane Handbook (38). For significant missing data, if the necessary information cannot be obtained from the literature, the original authors were contacted via email to request additional relevant data.

### 2.6 Statistical analysis

Data merging, heterogeneity testing, forest plots, and funnel plots were conducted using Stata 17.0 software. For the included literature, if the outcome indicators had consistent units or could be easily converted, the weighted mean difference (WMD) and its 95% confidence interval (CI) were used as the effect size for merging effects. If the units of the outcome indicators in the included literature were not uniform and could not be standardized through conversion, the standardized mean difference (SMD) and its 95% CI were used as the effect size for merging effects. In conducting the heterogeneity test, the Q statistic and I^2^ statistic were employed to assess the heterogeneity between studies. If the Q test results showed a p-value greater than 0.1 and an I^2^ value less than 50%, it indicates low heterogeneity among the studies, and a fixed-effects model was used for data merging. Conversely, if the Q test p-value was less than 0.1 or the I^2^ value was greater than 50%, it indicates significant heterogeneity, and a random-effects model was used for analysis (39). Funnel plots were created using Stata 17.0 to visually assess publication bias. Additionally, Egger’s test was used to quantify publication bias, with a significance level set at p < 0.05 (40, 41). Furthermore, sensitivity analyses were conducted to assess the stability of the results. This involved systematically removing individual studies, re-merging the effect sizes, and analyzing the data to observe changes in the merged results and to determine whether the results were influenced by any specific study or studies.

## 3 Results

### 3.1 Literature screening

**Figure 1:**
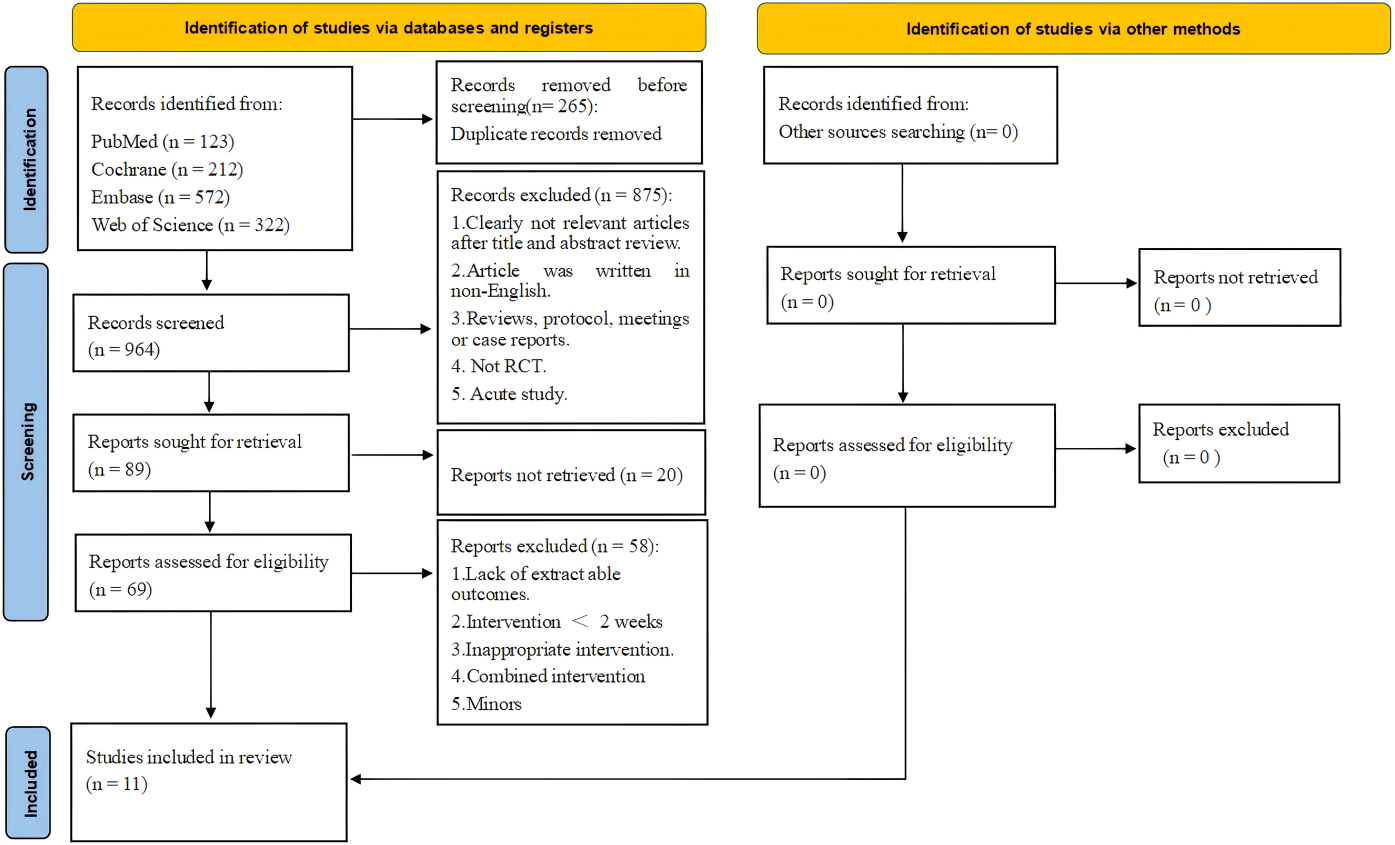
Flow diagram of systematic literature article search.

The literature search process is summarized in **Figure 1**. A precise search across four major databases (PubMed, Cochrane Library, Embase, and Web of Science) yielded a total of 1,229 relevant articles. During the initial screening of titles and abstracts, 1,160 articles that clearly did not meet the study’s topic or were duplicate publications were excluded, leaving 69 articles for full-text assessment. In the full-text assessment phase, strict screening was conducted using predefined inclusion and exclusion criteria. Ultimately, 58 articles were excluded for not meeting the study requirements, with the main exclusion reasons being the following: WBV training combined with other intervention methods, inability to obtain necessary research data, inclusion of underage subjects, intervention duration of less than 2 weeks, articles written in non-English languages, and other factors. Ultimately, 11 relevant RCTs were included in this systematic review and meta-analysis.

### 3.2 Basic information of included literature

The basic characteristics of the literature included in this study are summarized in **Table 1**. A total of 11 RCTs were included, involving 409 subjects, with 212 in the experimental group and 197 in the control group (42–52). All subjects in the included studies were adults, aged 18–85 years. Six studies included both male and female participants (43–45, 47, 49, 52); three studies included only female participants (42, 46, 51), while the remaining two did not report the gender ratio (48, 50). Regarding the population characteristics, three studies included patients with type 2 diabetes (43, 48, 50), and three studies included healthy older adults (44, 46, 52). Additionally, one study included healthy young women (42), one included patients with chronic obstructive pulmonary disease (45), one included patients with fatty liver disease (47), one included patients with interstitial lung disease (49), and one included women with premenstrual syndrome (51).

**Table 1:** Characteristics of included studies.

Authors	Population	Sample size (Exp/Con)	Sex (M/F)	Mean age (years)	Duration (weeks)	Frequency (times/week)	Amplitudes (mm)	Frequency (Hz)	Outcome measures	Intervention details (duration/sets/training volume)
Humphries et al., 2009 (42)	Healthy active women	9 9	0/9 0/9	18–30	12	2	1–6	50	TNF-α	Vibrate 30–60 s, rest 1 min/3 sets/gradually increase
Pozo-Cruz et al., 2014 (43)	Type 2 diabetes	19 20	8/11 10/10	71 66	12	3	4	12–16	TG, TC, LDL, HDL	Vibrate 30–60 s, rest 30 s/8 exercises/gradually increase
Rodriguez-Miguelez et al., 2015 (44)	Older adults	16 12	8/20	71 70	8	2	4	20–35	TNF-α, hs-CRP	Static or dynamic half-squat vibration
Neves et al., 2018 (45)	Patients with COPD	10 10	6/4 6/4	63 63	12	3	2	30–40	IL-6, IL-8	Vibrate 30 s, rest 1 min/6 sets/gradually increase
Piotrowska et al., 2018 (46)	Older adults	16 9	0/16 0/9	67	3	5	0.1–0.5	20–52	TG, TC, LDL, HDL	Vibration in bed for 30 min/2 sets
Oh et al., 2019 (47)	Patients with fatty liver disease	25 17	4/21 5/12	54 48	24	2	NR	30–50	TG	Divided into three training phases, a total of 20 min of vibration training
Domínguez-Muñoz et al., 2020 (48)	Type 2 diabetes	45 45	NR	40–85	8	3	NR	12.5–18.5	TC, LDL, HDL	Vibrate for 30–60 s, rest for 30 s/5–9 sets/gradually increase
Koczulla et al., 2020 (49)	Patients with interstitial lung disease	11 15	5/6 6/9	62 63	12	3	4–6	6–26	IL-6	Vibrate for 3 min, rest for 2 min/2–3 sets/gradually increase
Ramachandran et al., 2020 (50)	Type 2 diabetes	12 12	NR	51 50	12	3	2	30–35	TG, LDL, HDL	Vibrate for 20 min
Shehata et al., 2023 (51)	Women with premenstrual syndrome	35 35	0/35 0/35	21 22	12	3	1	14	hs-CRP	Vibrate for 1 min, rest for 1 min/1–10 sets/gradually increase
Timón et al., 2024 (52)	Older adults	14 13	6/8 6/7	>65	20	3	4	18–22	IL-8, TG, TC	Vibrate for 30 s, rest for 1 min/4 sets/gradually increase

Exp, experiment group; Con, control group; M, male, F, female; COPD, chronic obstructive pulmonary disease; mm, millimeter; Hz, hertz; TNF-α, tumor necrosis factor-alpha; IL, interleukin; hs-CRP, high-sensitivity C-reactive protein; TG, triglycerides; TC, total cholesterol; LDL, low-density lipoprotein; HDL, high-density lipoprotein; NR, not reported.

All studies employed simple WBV training interventions. In terms of intervention duration, six studies lasted for 12 weeks (42, 43, 45, 49–51), two lasted for 8 weeks (44, 48), one lasted 24 weeks (47), one lasted 20 weeks (52), and one lasted only 3 weeks (46). Seven studies conducted interventions three times a week (43, 45, 48–52), three studies conducted interventions twice a week (42, 44, 47), and one study conducted interventions five times a week (46). Most studies reported vibration amplitudes ranging from 1 to 6 mm (42–45, 49–52), one study reported amplitudes ranging from 0.1 to 0.5 mm (46), and two studies did not report specific vibration amplitudes (47, 48). All studies reported the vibration frequency. The specific intervention details are systematically summarized in **Table 1**.

Studies on inflammatory markers have indicated that interleukin-6 (IL-6) (48, 49) and interleukin-8 (IL-8) (45, 52) each had data from two studies, two studies reported tumor necrosis factor (TNF-α) (42, 44), and two provided data on high-sensitivity C-reactive protein (hs-CRP) (44, 51). In terms of lipid profile indicators, five studies provided data on triglycerides (TG) (43, 46, 47, 50, 52), and four studies provided data on total cholesterol (TC), LDL, and HDL (43, 46, 52). Therefore, based on the data from the included studies and existing authoritative literature (53, 54), this article considered IL-6, IL-8, TNF-α, and hs-CRP as inflammatory marker indicators, while TG, TC, LDL, and HDL were regarded as lipid profile indicators.

### 3.3 Meta-analysis results

#### 3.3.1 Effect of whole-body vibration training on interleukin-6 in adults

**Figure 2:**
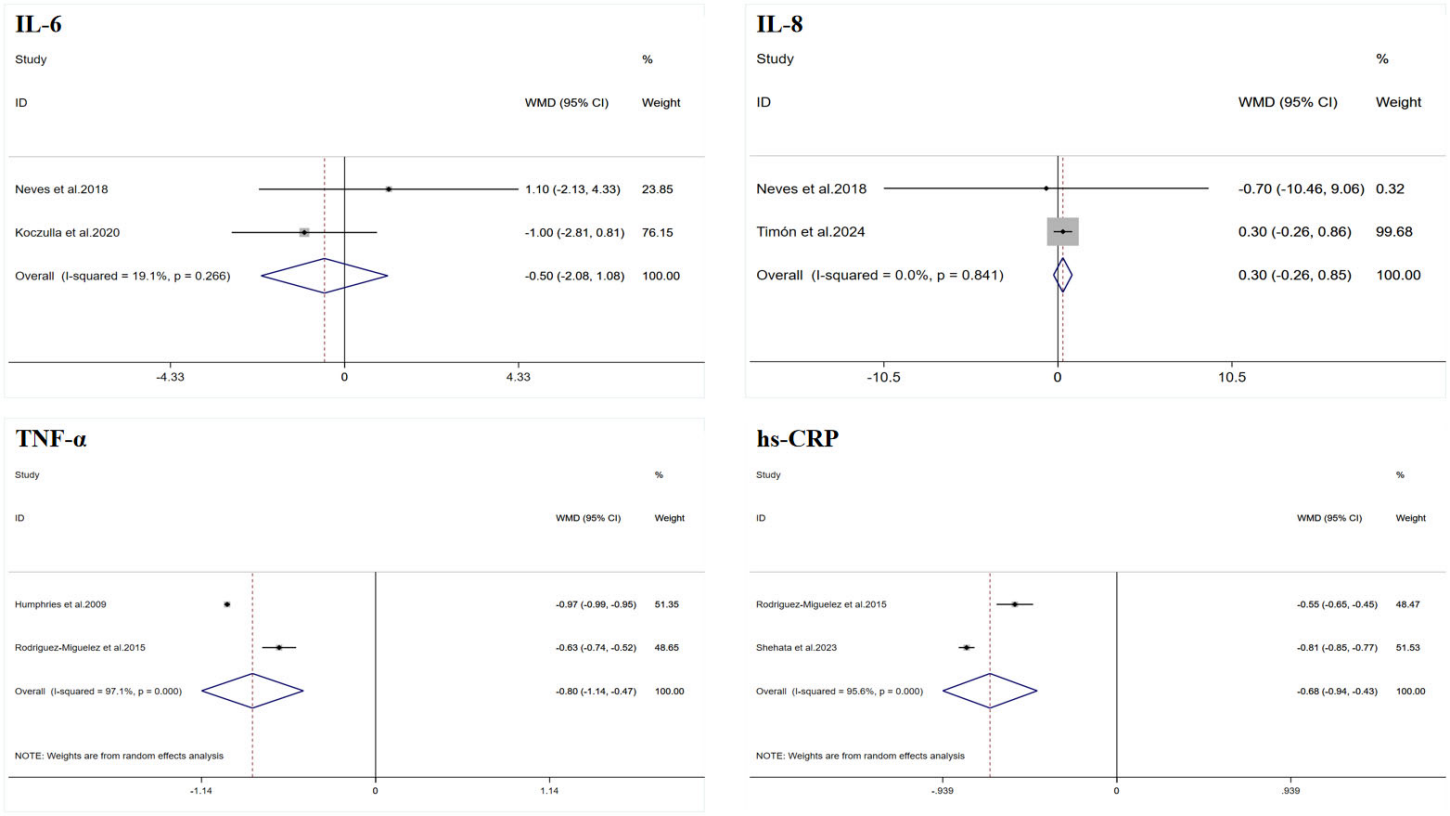
Forest plot of the effects of whole-body vibration training on inflammatory markers in adults. IL-6, interleukin-6; IL-8, interleukin-8; TNF-α, tumor necrosis factor-alpha; hs-CRP, high-sensitivity C-reactive protein.

A total of two datasets were included in the meta-analysis (**Figure 2**). The results of the meta-analysis showed that WBV training did not significantly improve IL-6 (WMD = −0.50 pg/mL, 95% CI: −2.08 to −1.08, p = 0.535), and the heterogeneity I^2^ was 19.1%, indicating that there was no significant heterogeneity between the study results (p = 0.226).

#### 3.3.2 Effect of whole-body vibration training on interleukin-8 in adults

A total of two datasets were included in the meta-analysis (**Figure 2**). The results of the meta-analysis showed that WBV training did not significantly improve IL-8 (WMD = 0.30 pg/mL, 95% CI: −0.26 to 0.85, p = 0.296), and the heterogeneity I^2^ was 0, indicating that there was no significant heterogeneity between the study results (p = 0.841).

#### 3.3.3 Effect of whole-body vibration training on tumor necrosis factor in adults

A total of two datasets were included in the meta-analysis (**Figure 2**). The results of the meta-analysis showed that WBV training significantly improved TNF-α (WMD = −0.80 pg/mL, 95% CI: −1.14 to −0.47, p < 0.001). However, there was a high degree of heterogeneity (I^2^ = 97.1%). Therefore, this result was summarized using a random-effects model for analysis.

#### 3.3.4 Effect of whole-body vibration training on high-sensitivity C-reactive protein in adults

A total of two datasets were included in the meta-analysis (**Figure 2**). The results of the meta-analysis showed that WBV training significantly improved hs-CRP (WMD = −0.68 mg/L, 95% CI: −0.94 to −0.43, p < 0.001). However, there was a high degree of heterogeneity (I^2^ = 95.6%). Therefore, this result was summarized using a random-effects model for analysis.

#### 3.3.5 Effect of whole-body vibration training on triglycerides in adults

**Figure 3:**
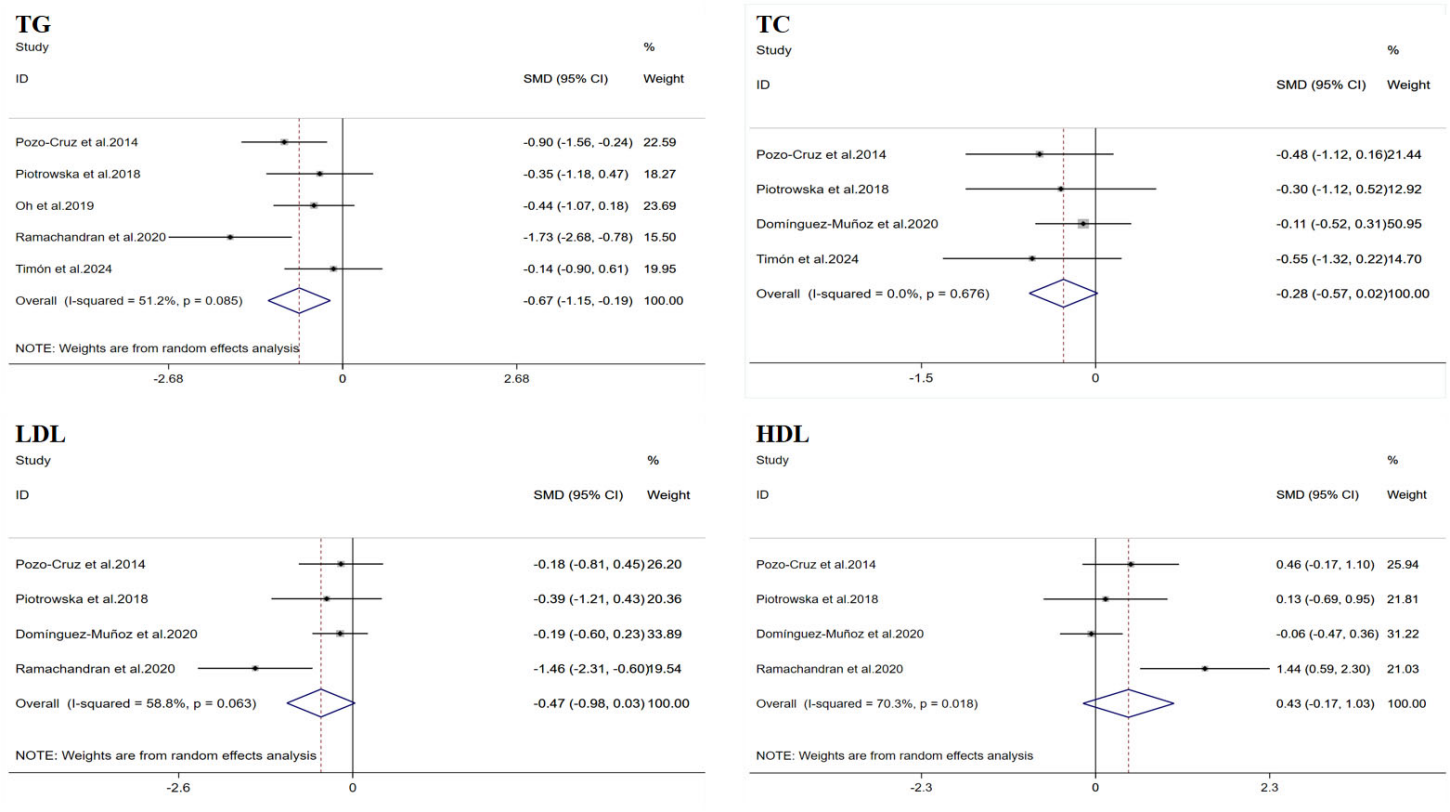
Forest plot of the effects of whole-body vibration training on lipid profiles in adults. TG, triglycerides; TC, total cholesterol; LDL, low-density lipoprotein; HDL, high-density lipoprotein.

A total of five datasets were included in the meta-analysis (**Figure 3**). Due to the inconsistency in the units provided by the studies, which could not be easily converted, SMD was used for the summary analysis. The results of the meta-analysis showed that WBV training significantly improved TG (SMD = −0.67, 95% CI: −1.15 to −0.19, p = 0.006). However, there was moderate heterogeneity (I^2^ = 51.2%). Therefore, this result was summarized using a random-effects model for analysis. Egger’s test indicated that there was no publication bias in the study results (p > 0.05).

#### 3.3.6 Effect of whole-body vibration training on total cholesterol in adults

A total of four datasets were included in the meta-analysis (**Figure 3**). Due to the inconsistency in the units provided by the studies, which could not be easily converted, SMD was used for the summary analysis. The results of the meta-analysis showed that WBV training did not significantly improve TC (SMD = −0.28, 95% CI: −0.57 to 0.02, p = 0.067), and the heterogeneity I^2^ was 0, indicating that there was no significant heterogeneity between the study results (p = 0.676). Egger’s test indicated that there was no publication bias in the study results (p > 0.05).

#### 3.3.7 Effect of whole-body vibration training on low-density lipoprotein in adults

A total of four datasets were included in the meta-analysis (**Figure 3**). Due to the inconsistency in the units provided by the studies, which could not be easily converted, SMD was used for the summary analysis. The results of the meta-analysis showed that WBV training did not significantly improve LDL (SMD = −0.47, 95% CI: −0.98 to 0.03, p = 0.068). However, there was moderate heterogeneity (I^2^ = 58.8%). Therefore, this result was summarized using a random-effects model for analysis. Egger’s test indicated that there was no publication bias in the study results (p > 0.05).

#### 3.3.8 Effect of whole-body vibration training on high-density lipoprotein in adults

A total of four datasets were included in the meta-analysis (**Figure 3**). Due to the inconsistency in the units provided by the studies, which could not be easily converted, SMD was used for the summary analysis. The results of the meta-analysis showed that WBV training did not significantly improve HDL (SMD = 0.43, 95% CI: −0.17 to 1.03, p = 0.156). However, there was a high degree of heterogeneity (I^2^ = 70.3%). Therefore, this result was summarized using a random-effects model for analysis. Egger’s test indicated that there was no publication bias in the study results (p > 0.05).

### 3.4 Risk of bias and literature quality assessment results

The risk of bias results indicated that Ramachandran et al. and Shehata et al. were rated as high risk in the “selection of the reported result” item due to the lack of clear measurement units for the data presented in the full text (50, 51); therefore, their overall risk was also assessed as high. The remaining studies were rated as low risk in all items.

The overall methodological quality of the included studies was high, with all literature classified as high-quality. Specifically, three studies received PEDro scores of 8 points (43, 49, 51), six studies scored 7 points (42, 44, 45, 47, 48, 52), and two studies scored 6 points (46, 50). The detailed results of the risk of bias and literature quality assessments are provided in **Supplementary Material 1**.

### 3.5 Publication bias tests and sensitivity analysis results

**Figure 4:**
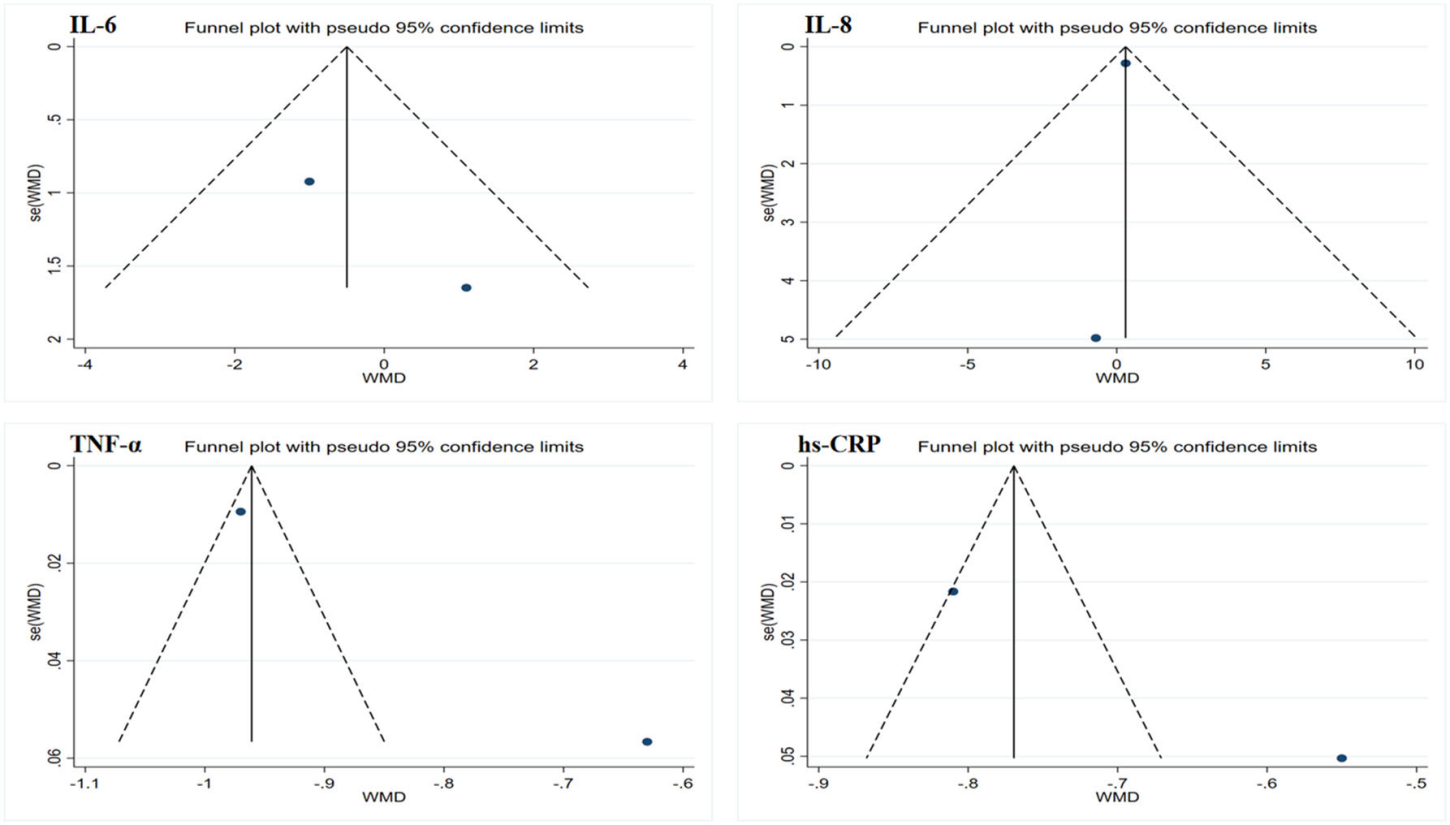
Funnel plot of publication bias for inflammatory markers. IL-6, interleukin-6; IL-8, interleukin-8; TNF-α, tumor necrosis factor-alpha; hs-CRP, high-sensitivity C-reactive protein.

**Figure 5:**
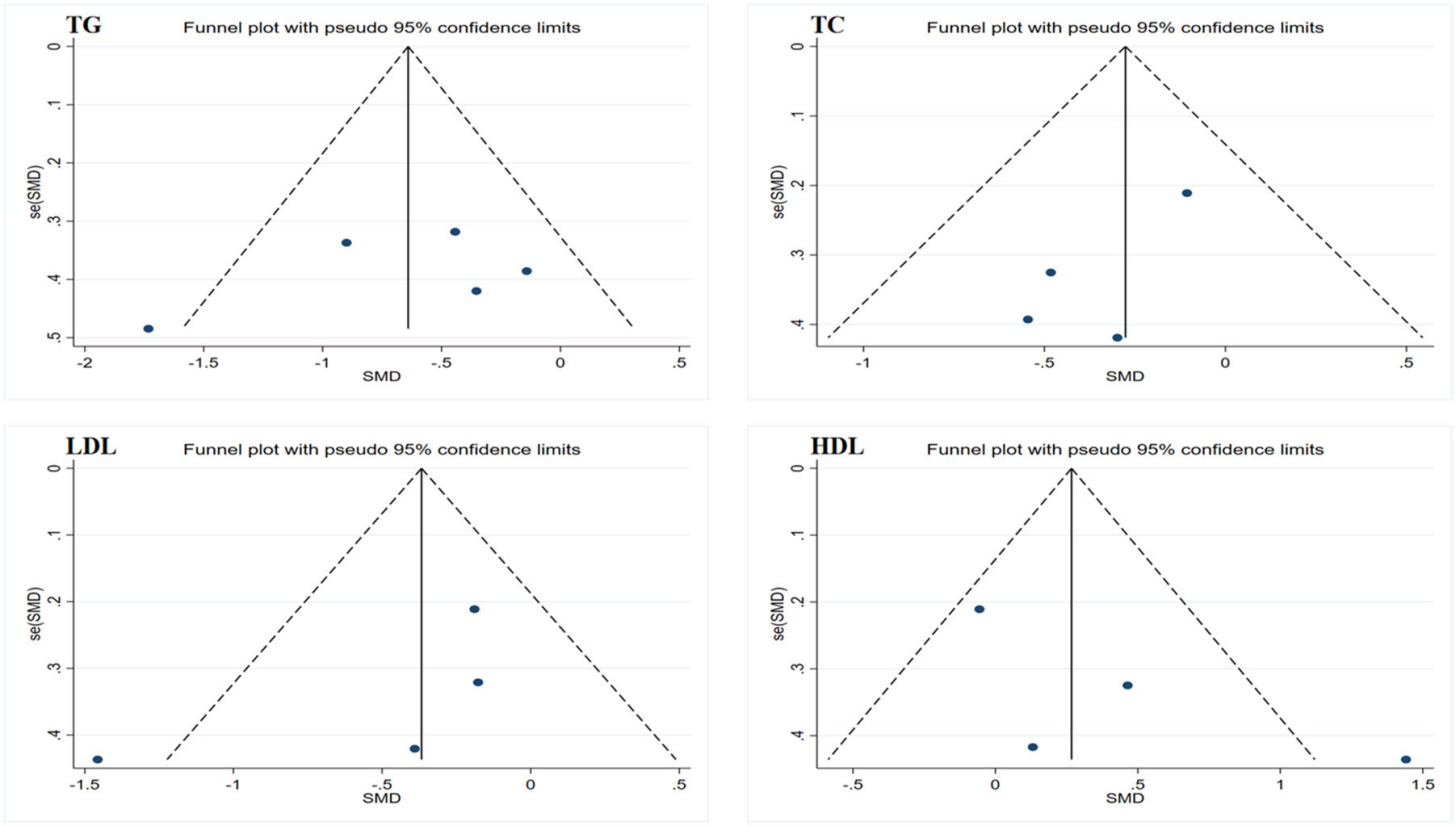
Funnel plot of publication bias for lipid profiles. TG, triglycerides; TC, total cholesterol; LDL, low-density lipoprotein; HDL, high-density lipoprotein.

Due to the limited sample size of the inflammation marker data, which consisted of only two studies, this research only conducted Egger’s test on the lipid profile results to assess publication bias. The results showed no evidence of publication bias. However, considering that all data sample sizes were less than 10, these results should be interpreted with caution. Funnel plots generated using Stata 17.0 software are summarized in **Figures 4** and **5**. Due to the sample size limitations, the results of the sensitivity analysis should also be approached with caution. Specifically, the sensitivity analysis showed that after excluding the results from Piotrowska et al., the heterogeneity among the TG data was eliminated, and the study results reached statistical significance (p < 0.05) (46). Moreover, after excluding the data from Ramachandran et al., the heterogeneity among the LDL and HDL data was eliminated; however, the study results still did not reach statistical significance (p > 0.05) (50). In summary, although no significant publication bias was found, the small sample size may affect the reliability of the results. Future studies should consider increasing the sample size to obtain more statistically significant conclusions.

## 4 Discussion

This systematic review integrated existing data from RCTs to investigate the effects of WBV training on inflammatory markers and lipid profile levels in adults. To our knowledge, this is the first meta-analysis based on RCTs examining the influence of WBV training on inflammatory markers and lipid profile levels in adults. The results indicate that WBV training can significantly improve inflammatory markers and lipid profile levels in adults, particularly showing notable improvements in TNF-α, hs-CRP, and TG levels. However, the existing evidence suggests that WBV training does not have a significant impact on IL-6, IL-8, TC, LDL, and HDL.

The results of this study indicate that WBV training has a significant positive impact on inflammatory markers (TNF-α and hs-CRP) and lipid profiles (TG) in adults, and this improvement has important clinical implications. Research has demonstrated that the levels of TNF-α, hs-CRP, and TG are closely associated with the occurrence of cardiovascular events. For instance, Tuomisto et al. found in a cohort study involving over 7,000 participants that individuals in the higher quartiles of TNF-α levels had a 2.09 times increased risk of cardiovascular events compared to those in the lowest quartile (55). However, a retrospective cohort study by Lee et al. involving 1,558 type 2 diabetes patients without CVD showed that subjects with hs-CRP levels above 0.21 mg/dL had a 1.77 times higher risk of major adverse cardiovascular events compared to those with hs-CRP levels below 0.08 mg/dL (56). Additionally, a meta-analysis by Patel et al. involving data from 96,224 participants revealed that after adjusting for major cardiovascular risk factors, individuals in the highest quintile of TG levels had a 70% increased risk of coronary heart disease mortality, an 80% increase in the risk of fatal or non-fatal coronary heart disease, and a 50% increase in the risk of fatal or non-fatal stroke, compared to those in the lowest quintile (57). Although large-scale studies quantifying the specific extent of improvement in TNF-α, hs-CRP, and TG levels in relation to the incidence of cardiovascular events and overall mortality are currently lacking, the clear associations between these markers and CVD risk underscore the clinical significance of WBV training. This finding highlights WBV training as an effective intervention that may reduce the incidence and mortality of CVD by lowering inflammation and improving lipid metabolism. An earlier review of Moreira-Marconi et al. indicated that WBV training may have potential benefits in managing inflammation for individuals with various clinical conditions (58). However, the study also emphasized the current lack of sufficient evidence to verify whether WBV training can significantly improve inflammation levels. Subsequently, Wang et al. conducted a meta-analysis incorporating experimental data related to both rodent and human studies, further confirming that WBV training may play a positive role in inflammation management for individuals in different clinical states (59). The findings of this study align with conclusions in the existing literature, further supporting the notion that WBV training can improve inflammation marker levels in adults, specifically leading to significant reductions in TNF-α and hs-CRP. Unlike the study published by Wang et al., our research established stricter inclusion and exclusion criteria, only incorporating RCTs that exclusively involved WBV training. This approach further substantiates the effectiveness of WBV training in improving inflammation markers by excluding the influence of confounding factors, thereby enhancing the credibility and clinical significance of the results. Although the mechanisms by which WBV training improves inflammation markers remain incompletely elucidated, WBV training, which stimulates all muscle groups through mechanical vibrations, may involve several underlying mechanisms. First, WBV training appears to enhance the production of NO by increasing shear stress (30, 60), which may help improve inflammation marker levels (61, 62). Research has demonstrated that NO can promote the polarization of pro-inflammatory macrophages (M1) to anti-inflammatory macrophages (M2) (63). Chow et al. conducted vibration therapy on 9-month-old ovariectomized osteoporotic rats and observed that the treatment restored the number of M2 macrophages to normal levels while facilitating the transition from inflammatory M1 to anti-inflammatory M2 (64). Additionally, Yu et al. implemented a 4-week WBV intervention on diabetic mice, resulting in M2 levels in these mice approaching baseline levels of normal controls (65). However, current studies have not clearly defined the direct relationship between M2 macrophage polarization induced by WBV training and the NO generated from WBV, and there is a lack of related human experiments; thus, interpretations of these results should be approached with caution. Second, WBV training appears to inhibit the activation of nuclear factor kappa B (NF-κB), thereby alleviating inflammatory responses. Rodriguez-Miguelez et al. conducted an 8-week WBV training program on elderly individuals, revealing that WBV training may downregulate the expression of tumor necrosis factor receptor-associated factor 4 (TLR-4) and 2 (TLR-2), mediated by myeloid differentiation primary response gene 88 (MyD88), thus subsequently downregulating NF-κB activation (44). Third, WBV training significantly reduces inflammation levels, likely related to improvements in plasma levels of soluble tumor necrosis factor-α receptors 1 (sTNFR1) and 2 (sTNFR2). Studies have indicated that sTNFR1 is closely associated with pro-inflammatory responses and apoptosis (66), while sTNFR2 is tightly linked to various immunoregulatory and anti-inflammatory functions (67). *In vivo*, a complex interplay, including synergistic and antagonistic effects, exists between sTNFR1 and sTNFR2 (68, 69). Evidence from Simao et al. showed that a 12-week WBV training program in elderly patients with knee osteoarthritis resulted in significantly lower levels of sTNFR1 and sTNFR2 in the WBV training group compared to two other groups (70). Furthermore, the results of Almeida et al. from a one-time acute WBV training program in elderly sarcopenic and non-sarcopenic patients indicated that the training could significantly elevate sTNFR2 levels in both groups (71). Finally, WBV training can improve inflammation levels, which may correlate with its ability to modify the levels of various cytokines in the body. For instance, Wang et al. administered a 2-month low-frequency high-amplitude vibration intervention to mice with muscle atrophy, and the results indicated a significant increase in adiponectin levels in this group (72). Notably, this substantial increase in adiponectin levels has been observed not only in animal studies but also in a 16-week study of healthy active women conducted by Humphries et al. (42). Subsequent research has also confirmed a correlation between WBV training and elevated adiponectin levels (31, 47). Moreover, multiple studies have demonstrated that WBV training can significantly enhance the levels of irisin in humans (32, 70). Current evidence has suggested that both adiponectin and irisin play crucial roles in anti-inflammatory mechanisms (73). Both substances contribute not only in anti-inflammatory processes but also in enhancing lipid metabolism, improving insulin sensitivity, and reducing fat accumulation, thereby optimizing adipose tissue function and lowering the risk of obesity and related diseases (74, 75). Consequently, this paper also observed that WBV training can significantly improve TG levels in adults. Although the specific mechanisms through which WBV training improves lipid profiles remain unclear, it likely impacts TG levels through interactions across various physiological systems, including skeletal, muscular, endocrine, neural, and vascular systems. First, as mentioned in the text, WBV training significantly elevates the levels of both adiponectin and irisin, which play key roles in regulating lipid metabolism. Second, studies have indicated that participating in WBV training can significantly increase muscle temperature (76), blood flow (77), and oxygen consumption (78), a process that may activate adenosine monophosphate-activated protein kinase (AMPK)-related signaling pathways and subsequently improve mitochondrial function, thus aiding in the reduction of TG levels (79). Research has indicated that during WBV training, the expression of AMPK in skeletal muscle significantly increases (79). AMPK acts as an energy sensor in skeletal muscle, reducing the production of malonyl-CoA by phosphorylating and inactivating acetyl-CoA carboxylase β (ACCβ) (80). Malonyl-CoA is a potent inhibitor of carnitine palmitoyltransferase I (CPT1) (81). CPT1 is a key rate-limiting enzyme located in the outer mitochondrial membrane, responsible for the oxidation of fatty acids, directly promoting the process of fatty acid β-oxidation (82, 83). Existing evidence generally supports that AMPK signaling through the ACC–malonyl-CoA–CPT1 pathway is an important mechanism for regulating fatty acid oxidation in skeletal muscle during exercise (84). Furthermore, acute WBV exposure can activate the sympathetic nervous system (SNS) (85, 86), one major function of which is to promote lipolysis in white adipose tissue (87). Studies have shown that the balance of catecholamine and calcium ion levels within muscle cells, along with the energy status, determines the overall activation state of hormone-sensitive lipase (HSL) and its potential for lipolysis in skeletal muscle during exercise (88). As a key molecule in the lipolytic process, the activity of HSL is influenced by catecholamine levels in the body (89). The stimulation from WBV training can directly activate the SNS, leading to the release of norepinephrine, which promotes the phosphorylation and activation of HSL by binding to receptors on adipocytes, thus triggering the breakdown of fats. In summary, combining the existing evidence, it can be inferred that WBV training enhances fatty acid oxidation in skeletal muscle not only through the AMPK-ACC–malonyl-CoA–CPT1 signaling pathway but also by activating the SNS and promoting HSL phosphorylation, effectively improving the overall efficiency of fat metabolism.

Despite existing studies indicating that WBV training can positively affect inflammation markers and lipid profiles through various mechanisms, this study, after integrating current evidence, found that WBV training did not significantly improve IL-6, IL-8, TC, LDL, and HDL levels in adults. This result can primarily be attributed to several factors. First, the different WBV training programs employed in various studies may have influenced the outcomes. For instance, the 12-week WBV training conducted by Neves et al. on patients with moderate chronic obstructive pulmonary disease, using an amplitude of 2 mm and a frequency of 30–40 Hz, showed that there was no significant improvement in IL-6 levels in the intervention group (45). However, the same length of WBV training conducted by Koczulla et al. on patients with interstitial lung disease, using a program with an amplitude of 4–6 mm and a frequency of 6–26 Hz, revealed a reduction of −1 pg/mL in serum IL-6 levels in the intervention group, while the control group remained unchanged (49). Thus, differences in factors such as amplitude and frequency may account for the inconsistencies in the results across studies. In addition, the participants included in this study were all adults, but the groups involved consisted of patients with various conditions, such as diabetes, chronic obstructive pulmonary disease, fatty liver disease, and interstitial lung disease, which may also lead to differences in study results and affect the general applicability of the findings. Finally, the relatively small number of studies included led to a limited sample size, which overall impacted the results of the statistical analyses. Among the only four studies that provided TC data, three explicitly stated that a significant improvement in TC was observed after WBV training (43, 46, 48). Meanwhile, although the statistical results for TC, LDL, and HDL did not reach significance, there was an overall trend of improvement. Thus, the limitation of sample size is also an important factor affecting these results. Despite these differences, this meta-analysis confirms that WBV training can significantly improve TNF-α, hs-CRP, and TG levels in adults. Future research should design different group comparative RCTs targeting the same population to validate the relationship between varying training parameters, such as vibration frequency, amplitude, and duration, with these mechanisms, thereby providing stronger evidence for optimizing WBV training protocols.

## 5 Clinical significance and future perspectives

Chronic inflammation and dyslipidemia are critical pathological mechanisms in the development of CVD. This study confirms that WBV training significantly enhances inflammation markers (TNF-α and hs-CRP) and lipid metabolism levels (TG) in adults, providing important evidence-based clinical support for non-pharmacological prevention and treatment of CVD. WBV training, as a non-pharmacological intervention strategy, offers a safe, effective, and easy-to-implement exercise alternative, particularly for special populations who may struggle to endure traditional moderate- to high-intensity exercise due to aging, chronic diseases, or functional limitations. None of the clinical trials included in this research reported adverse events; the study by Neves et al. noted that only two participants withdrew due to personal interest (45), indicating good overall adherence to the intervention and suggesting that WBV training has good acceptability and safety. Future studies could design interventions that combine WBV training with other methods (e.g., dietary interventions or other forms of exercise), as multiple studies have shown that comprehensive intervention strategies exhibit certain advantages in promoting health and improving various CVD risk factors (30, 31, 90, 91). Additionally, incorporating more engaging and personalized elements into WBV training (such as music) could enhance participant adherence to training with the aim of maximizing the health benefits of WBV training.

## 6 Limitations

This study has some inevitable limitations. First, this study only included 11 RCTs, and the data on relevant inflammation markers were insufficient, which may have some impact on the strength of the evidence for the statistical results. Second, the limited sample size restricts our ability to conduct in-depth subgroup analyses of WBV training parameters (such as vibration frequency, amplitude, and training duration) and population characteristics (such as age, gender, and health status). Consequently, the optimal training regimen cannot be determined, making it challenging to assess differences in effects under varied intervention conditions. Finally, due to the small number of studies included, the statistical power of Egger’s test was limited and may not have been sufficient to adequately identify potential publication bias, which, to some extent, restricted the evidence quality of the study results. More large-scale RCTs are necessary in the future to further elucidate the impact of WBV training on inflammation markers and lipid profile levels while exploring the underlying biological mechanisms and their relationship with overall health and disease prevention.

## 7 Conclusion

This study confirms that WBV training can positively influence inflammation markers and lipid profiles in adults, specifically demonstrating significant reductions in TNF-α, hs-CRP, and TG levels. These findings provide essential support for health management and intervention measures aimed at chronic disease prevention in adults, further reinforcing the potential of WBV training as an effective exercise intervention strategy in promoting metabolic health.

## Data Availability

The original contributions presented in the study are included in the article/**Supplementary Material**. Further inquiries can be directed to the corresponding author.
