# Supplementary material for: Effects of whole-body vibration training on inflammatory markers and lipid profiles in adults: a systematic review and meta-analysis of randomized controlled trials
Source: Front Immunol. 2025 Nov 10;16:1705866. doi: 10.3389/fimmu.2025.1705866 (PMC12640864; doi:10.3389/fimmu.2025.1705866)

**Supplementary Table 1. Risk of Bias Assessment using RoB 2**

| **Study lD** | **Randomisation process** | **Deviations from the intended interventions** | **Mising outcome data** | **Measurement of the outcome** | **Selection of the reported result** | **Overall risk-of-bias judgment** |
| --- | --- | --- | --- | --- | --- | --- |
| Humphries et al.  2009 | Low risk | Low risk | Low risk | Low risk | Low risk | Low risk |
| Pozo-Cruz et al.  2014 | Low risk | Low risk | Low risk | Low risk | Low risk | Low risk |
| Rodriguez-Miguelez et al.2015 | Low risk | Low risk | Low risk | Low risk | Low risk | Low risk |
| Neves et al. 2018 | Low risk | Low risk | Low risk | Low risk | Low risk | Low risk |
| Piotrowska et al.  2018 | Low risk | Low risk | Low risk | Low risk | Low risk | Low risk |
| Oh et al.2019 | Low risk | Low risk | Low risk | Low risk | Low risk | Low risk |
| Domínguez-Muñoz et al.2020 | Low risk | Low risk | Low risk | Low risk | Low risk | Low risk |
| Koczulla et al.  2020 | Low risk | Low risk | Low risk | Low risk | Low risk | Low risk |
| Ramachandran et al.2020 | Low risk | Low risk | Low risk | Low risk | High risk | High risk |
| Shehata et al.  2023 | Low risk | Low risk | Low risk | Low risk | High risk | High risk |
| Timón et al.  2024 | Low risk | Low risk | Low risk | Low risk | Low risk | Low risk |

**Supplementary Table 2. literature quality assessment**

| **PEDro score** | | | | | | | | | | | | |
| --- | --- | --- | --- | --- | --- | --- | --- | --- | --- | --- | --- | --- |
|  | **Eligibility criteria** | **Random**  **allocation** | **Concealed**  **allocation** | **Similar**  **baseline** | **Participant**  **blinding** | **Investigator blinding** | **Assessor**  **blinding** | **Completeness of follow-up** | **Intention to treat** | **Between-group comparisons** | **Point measures and variability** | **Total score** |
| Humphries et al.  2009 | 1 | 1 | 0 | 1 | 0 | 0 | 0 | 1 | 1 | 1 | 1 | 7 |
| Pozo-Cruz et al.  2014 | 1 | 1 | 1 | 1 | 0 | 0 | 0 | 1 | 1 | 1 | 1 | 8 |
| Rodriguez-Miguelez et al.2015 | 1 | 1 | 0 | 1 | 0 | 0 | 0 | 1 | 1 | 1 | 1 | 7 |
| Neves et al. 2018 | 1 | 1 | 0 | 1 | 0 | 0 | 0 | 1 | 1 | 1 | 1 | 7 |
| Piotrowska et al.  2018 | 1 | 1 | 0 | 1 | 0 | 0 | 0 | 0 | 1 | 1 | 1 | 6 |
| Oh et al.2019 | 1 | 1 | 0 | 1 | 0 | 0 | 0 | 1 | 1 | 1 | 1 | 7 |
| Domínguez-Muñoz et al.2020 | 1 | 1 | 0 | 1 | 0 | 0 | 0 | 1 | 1 | 1 | 1 | 7 |
| Koczulla et al.  2020 | 1 | 1 | 1 | 1 | 0 | 0 | 0 | 1 | 1 | 1 | 1 | 8 |
| Ramachandran et al.2020 | 1 | 1 | 0 | 1 | 0 | 0 | 0 | 0 | 1 | 1 | 1 | 6 |
| Shehata et al.  2023 | 1 | 1 | 1 | 1 | 0 | 0 | 0 | 1 | 1 | 1 | 1 | 8 |
| Timón et al.  2024 | 1 | 1 | 0 | 1 | 0 | 0 | 0 | 1 | 1 | 1 | 1 | 7 |

**Supplementary Table 3. Search strategy**

| **1.PubMed: 123** |
| --- |
| Set1: ((((whole-body vibration training[Title/Abstract]) OR (whole body vibration training[Title/Abstract])) OR (vibration training[Title/Abstract])) OR (vibration[Title/Abstract])) OR (WBVT[Title/Abstract]) |
| Set2: ("Inflammation"[Mesh]) OR ((inflammatory response[Title/Abstract]) OR (inflammatory markers[Title/Abstract])) |
| Set3: ("Interleukins"[Mesh]) OR (IL[Title/Abstract]) |
| Set4: ("Tumor Necrosis Factor-alpha"[Mesh]) OR ((TNF-alpha[Title/Abstract]) OR (TNF-α[Title/Abstract])) |
| Set5: ("C-Reactive Protein"[Mesh]) OR (((CRP[Title/Abstract]) OR (high sensitivity C-reactive protein[Title/Abstract])) OR (hs-CRP[Title/Abstract])) |
| Set6: ("Lipids"[Mesh]) OR (((((lipid profile[Title/Abstract]) OR (total cholesterol[Title/Abstract])) OR (TC[Title/Abstract])) OR (triglycerides[Title/Abstract])) OR (TG[Title/Abstract])) |
| Set7: ("Lipoproteins"[Mesh]) OR ((((((((((low-density lipoprotein[Title/Abstract]) OR (LDL[Title/Abstract])) OR (low-density lipoprotein cholesterol[Title/Abstract])) OR (LDL cholesterol[Title/Abstract])) OR (LDL-C[Title/Abstract])) OR (high-density lipoprotein[Title/Abstract])) OR (HDL[Title/Abstract])) OR (high-density lipoprotein cholesterol[Title/Abstract])) OR (HDL cholesterol[Title/Abstract])) OR (HDL-C[Title/Abstract])) |
| Set8: (((((((random[Title/Abstract]) OR (randomized[Title/Abstract])) OR (randomly[Title/Abstract])) OR (randomised[Title/Abstract])) OR (randomized controlled trial[Title/Abstract])) OR (RCT[Title/Abstract])) OR (randomized controlled trials[Title/Abstract])) OR (RCTs[Title/Abstract]) |
| Set9: #2 OR #3 OR #4 OR #5 OR #6 OR #7 |
| Set10: #1 AND #8 AND #9 |
|  |
| **2.Cochrane: 212** |
| Set1: MeSH descriptor: [Inflammation] explode all trees |
| Set2: MeSH descriptor: [Interleukins] explode all trees |
| Set3: MeSH descriptor: [Tumor Necrosis Factor-alpha] explode all trees |
| Set4: MeSH descriptor: [C-Reactive Protein] explode all trees |
| Set5: MeSH descriptor: [Lipids] explode all trees |
| Set6: MeSH descriptor: [Lipoproteins] explode all trees |
| Set7: (inflammatory response):ti,ab,kw or (inflammatory markers):ti,ab,kw |
| Set8: (interleukins):ti,ab,kw or (inflammatory markers):ti,ab,kw |
| Set9: (TNF-alpha):ti,ab,kw or (TNF-α):ti,ab,kw |
| Set10: CRP):ti,ab,kw or (high sensitivity C-reactive protein):ti,ab,kw or (hs-CRP):ti,ab,kw |
| Set11: (lipid profile):ti,ab,kw or (total cholesterol):ti,ab,kw or (TC):ti,ab,kw or (triglycerides):ti,ab,kw or (TG):ti,ab,kw |
| Set12: (low-density lipoprotein):ti,ab,kw or (LDL):ti,ab,kw or (low-density lipoprotein cholesterol):ti,ab,kw or (LDL cholesterol):ti,ab,kw or (LDL-C):ti,ab,kw or (high-density lipoprotein):ti,ab,kw or (HDL):ti,ab,kw or (high-density lipoprotein cholesterol):ti,ab,kw or (HDL cholesterol):ti,ab,kw or (HDL-C):ti,ab,kw |
| Set13: (whole-body vibration training):ti,ab,kw or (whole body vibration trainin):ti,ab,kw or (vibration training):ti,ab,kw or (vibration):ti,ab,kw or (WBVT):ti,ab,kw |
| Set14: #1 OR #2 OR #3 OR #4 OR #5 OR #6 OR #7 OR #8 OR #9 OR #10 OR #11 OR #12 |
| Set15: #13 AND #14 |
|  |
| **3.Embase: 572** |
| Set1: 'inflammation'/exp |
| Set2: 'interleukin'/exp |
| Set3: 'tumor necrosis factor alpha'/exp |
| Set4: 'c reactive protein'/exp |
| Set5: 'lipid'/exp |
| Set6: 'lipoprotein'/exp |
| Set7: 'citrulline malate':ab,ti OR 'l-citrulline':ab,ti |
| Set8: 'inflammatory response':ab,ti OR 'inflammatory markers':ab,ti |
| Set9: 'il':ab,ti |
| Set10: 'il':ab,ti |
| Set11: 'crp':ab,ti OR 'high sensitivity c-reactive protein':ab,ti OR 'hs-crp':ab,ti |
| Set12: 'lipid profile':ab,ti OR 'total cholesterol':ab,ti OR 'tc':ab,ti OR 'triglycerides':ab,ti OR 'tg':ab,ti |
| Set13: 'low-density lipoprotein':ab,ti OR 'ldl':ab,ti OR 'low-density lipoprotein cholesterol':ab,ti OR 'ldl cholesterol':ab,ti OR 'ldl-c':ab,ti OR 'high-density lipoprotein':ab,ti OR 'hdl':ab,ti OR 'high-density lipoprotein cholesterol':ab,ti OR 'hdl cholesterol':ab,ti OR 'hdl-c':ab,ti |
| Set14: 'whole-body vibration training':ab,ti OR 'whole body vibration trainin':ab,ti OR 'vibration training':ab,ti OR 'vibration':ab,ti OR 'wbvt':ab,ti |
| Set15: 'random':ab,ti OR 'randomized':ab,ti OR 'randomly':ab,ti OR 'randomised':ab,ti OR 'randomized controlled trial':ab,ti OR 'rct':ab,ti |
| Set16: #1 OR #2 OR #3 OR #4 OR #5 OR #6 OR #7 OR #8 OR #9 OR #10 OR #11 OR #12 OR #13 |
| Set17: #14 AND #15 AND #16 |
|  |
| **4.Web of science：185** |
| Set1: whole-body vibration training (Topic) or whole body vibration trainin (Topic) or vibration training (Topic) or vibration (Topic) or WBVT (Topic) |
| Set2: inflammation (Topic) or inflammatory response (Topic) or inflammatory markers (Topic) or interleukins (Topic) or IL (Topic) or tumor necrosis factor alpha (Topic) or TNF-alpha (Topic) or TNF-α (Topic) or C-reactive protein (Topic) or CRP (Topic) or high sensitivity C-reactive protein (Topic) or hs-CRP (Topic) or lipids (Topic) or lipid profile (Topic) or total cholesterol (Topic) or TC (Topic) or triglycerides (Topic) or TG (Topic) or lipoproteins (Topic) or low-density lipoprotein (Topic) or LDL (Topic) or low-density lipoprotein cholesterol (Topic) or LDL cholesterol (Topic) or LDL-C (Topic) or high-density lipoprotein (Topic) or HDL (Topic) or high-density lipoprotein cholesterol (Topic) or HDL cholesterol (Topic) or HDL-C (Topic) |
| Set3: random (Topic) or randomized (Topic) or randomly (Topic) or randomised (Topic) or randomized controlled trial (Topic) or RCT (Topic) or randomized controlled trials (Topic) or RCTs (Topic) |
| Set4: 1# AND #2 AND #3 |


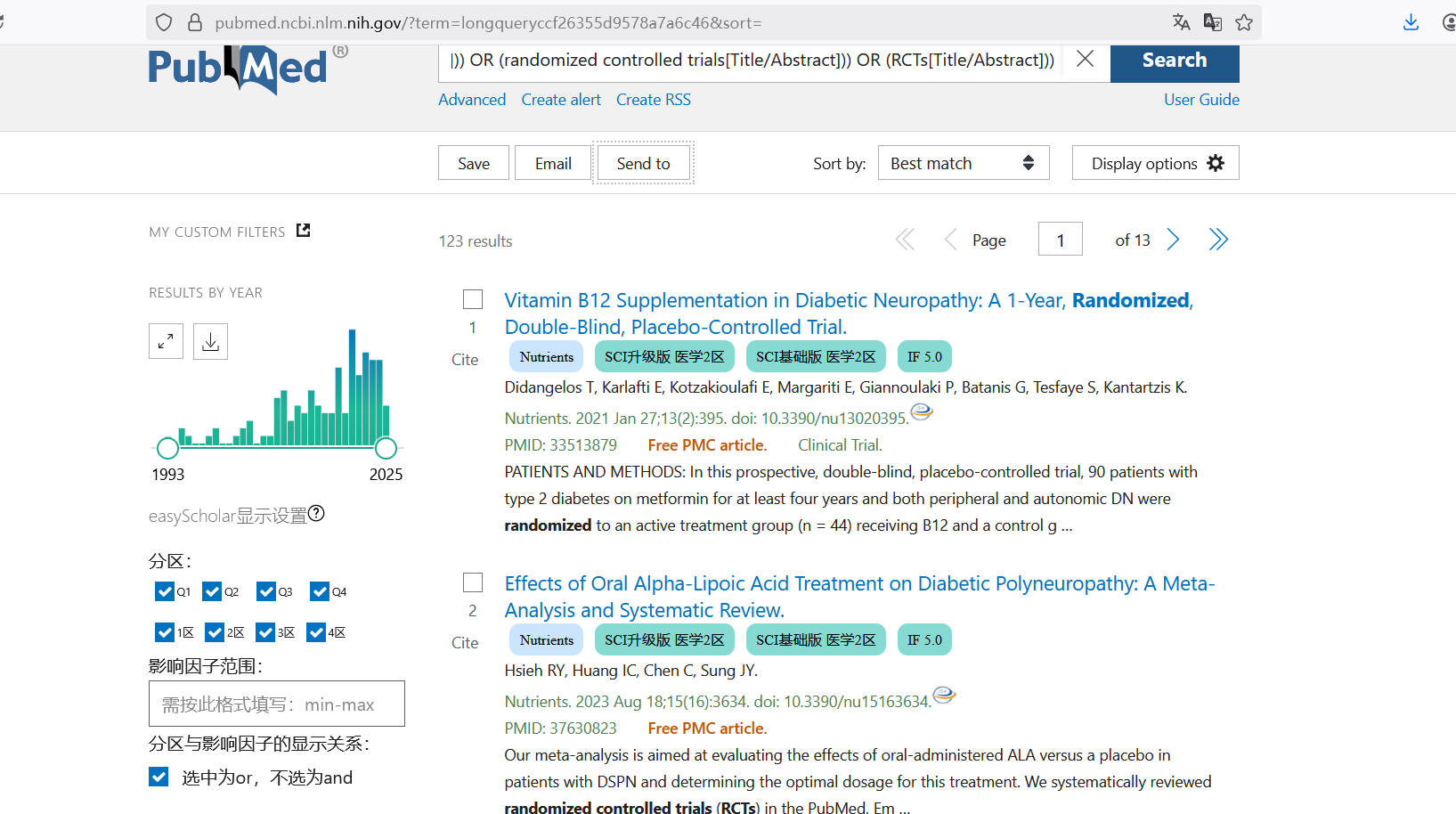


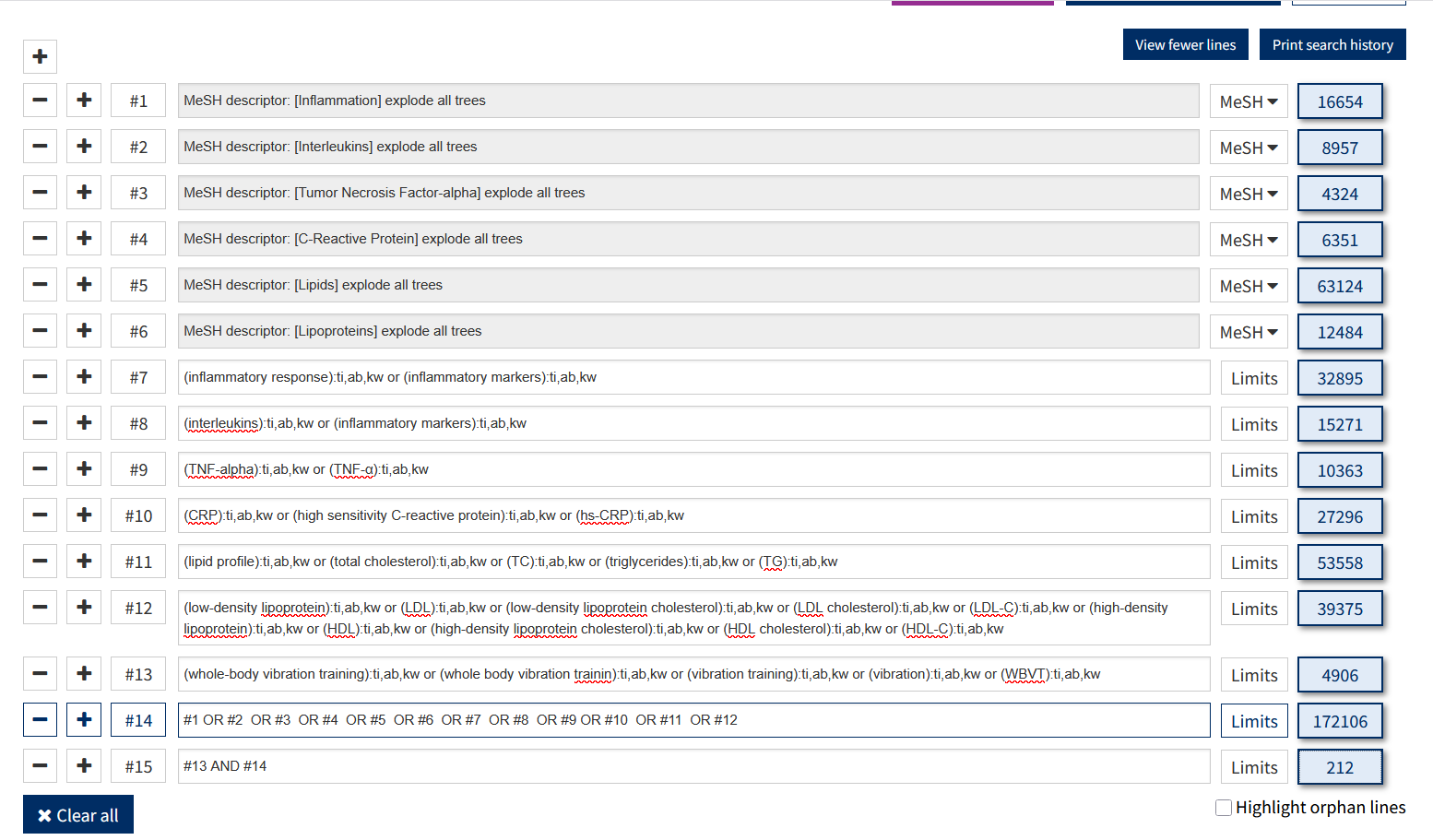


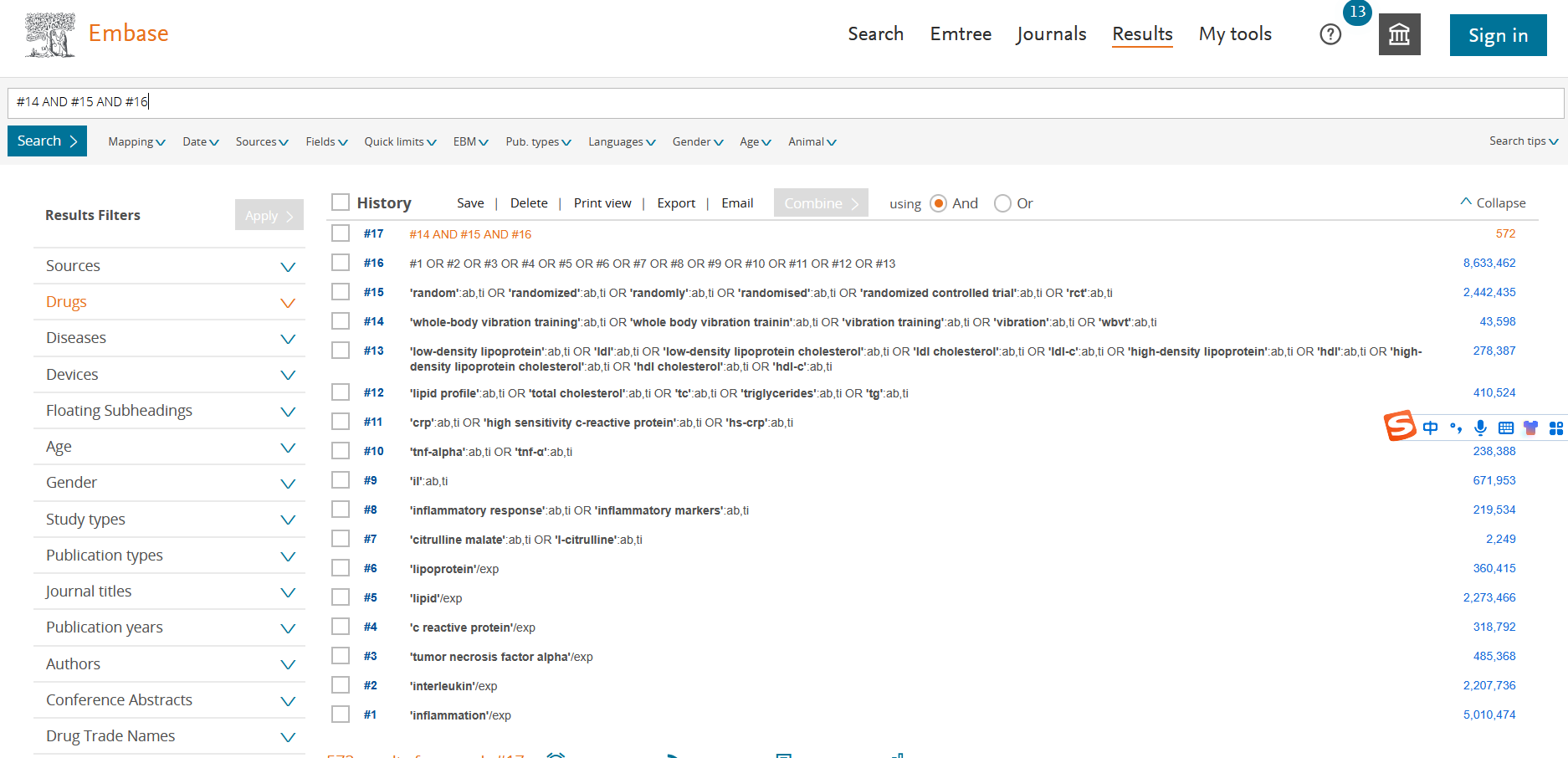


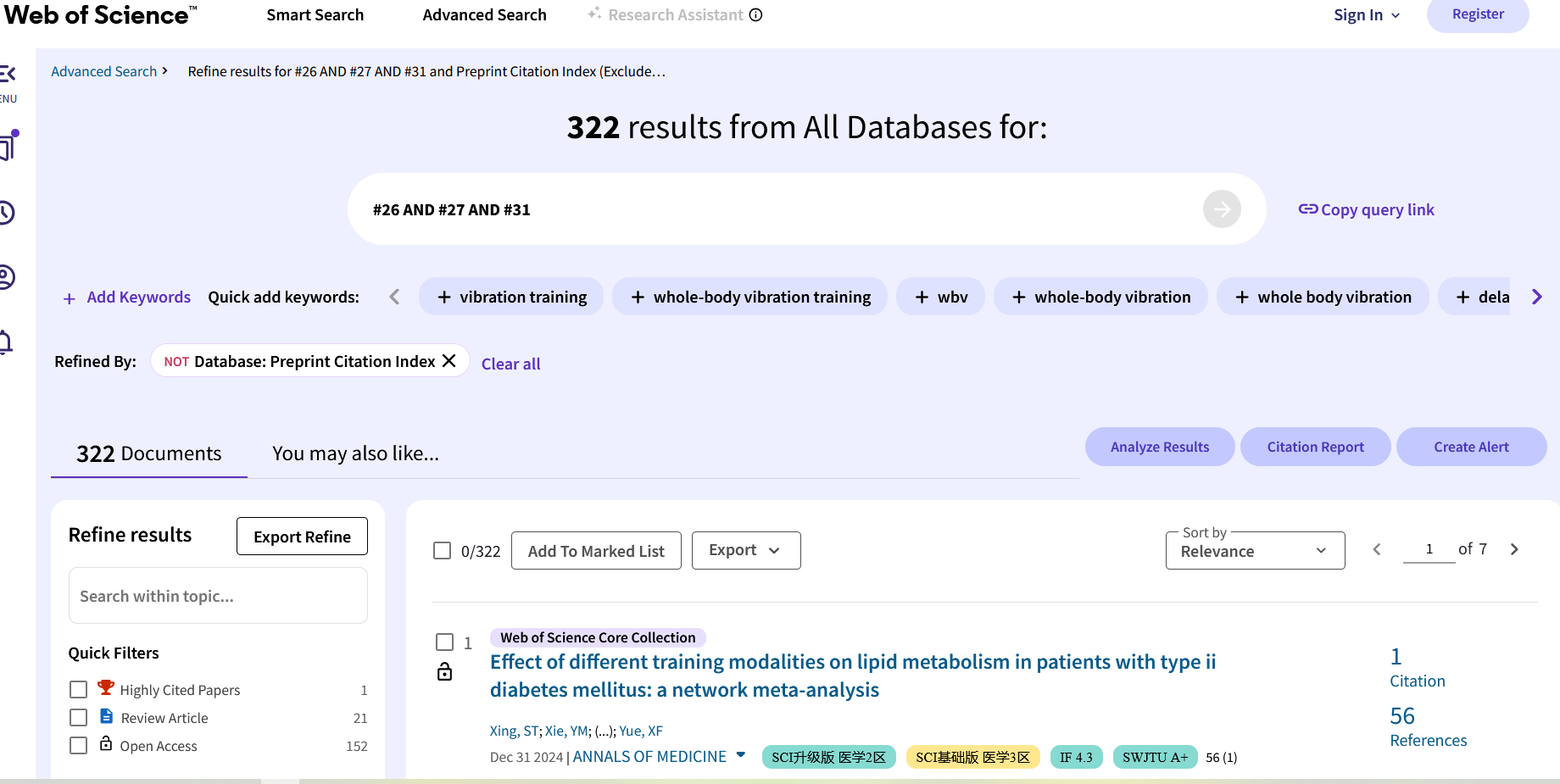

Supplement: Supplementary file 1 [file DataSheet1.docx]
